# The association between depression and thyroid function

**DOI:** 10.3389/fendo.2024.1454744

**Published:** 2024-08-30

**Authors:** Yuhui Ma, Miao Wang, Zhishen Zhang

**Affiliations:** ^1^ Department of Ultrasound, Qilu Hospital (Qingdao), Cheeloo College of Medicine, Shandong University, Qingdao, China; ^2^ Department of Office, Qingdao Chest Hospital, Qingdao, China; ^3^ Department of Psychological, Qingdao Central Hospital, University of Health and Rehabilitation Sciences (Qingdao Central Hospital), Qingdao, China

**Keywords:** thyroid, thyroid function, depression, NHANES, cross-section study, PHQ-9

## Abstract

**Background:**

Emerging evidence indicated that depression is currently one of the most burdensome diseases worldwide, and it can lead to a variety of functional physical impairments. However, the studies estimated the association between depression and thyroid function remain sparse. We aimed to investigate the association between depression and thyroid function in the American population.

**Methods:**

A cross-sectional analysis was performed using the data from the National Health and Nutrition Examination Survey conducted from 2007 to 2012. In the 12,502 adults aged 20–80 years, weighted linear regression models and multiple logistic regression models were applied to evaluate the association between depression and thyroid function indicators. The thyroid indicators investigated were mainly free thyroxine (FT4), total T4 (TT4), free triiodothyronine (FT3), total T3 (TT3), thyroid-stimulating hormone (TSH), and antithyroperoxidase antibody (TPOAb), thyroglobulin (Tg) and antithyroglobulin antibody (TgAb).

**Results:**

The final results were reached after adjusting for various confounding factors. In the stratification analysis of subgroups divided by age, depression was significantly negatively correlated with FT4, FT3, and TT3 in both younger adults (*p* = 0.00122, *p* < 0.00001, and *p* = 0.00003) and older adults (*p* = 0.00001, *p* = 0.00004, and *p* < 0.00001). In contrast, depression was significantly negatively correlated with TT4 and Tg in older adults (p = 0.00054, p = 0.00695) and positively correlated in younger adults (p = 0.01352, p < 0.00001). The subgroup analysis by gender revealed that depression was significantly negatively correlated with FT4, FT3, and TT3 in both adult males (*p* = 0.0164, *p* = 0.0204, and *p* = 0.0050) and adult females (*p* ≤ 0.0001, *p* < 0.0001, and *p* < 0.0001), which was more prominent in females. The positive correlation between depression symptoms and TPOAb was only found in adult females (p = 0.0282) and younger adults (p = 0.00488).

**Conclusion:**

This study confirmed a significant correlation between depressive and thyroid function and it varied among different genders or age. In the future, more prospective studies are needed to reveal these findings and confirm a causal relationship between them.

## 1 Introduction

Depression is a mood disorder that can lead to a variety of functional physical impairments and loss of interest in daily activities, which can reduce quality of life (1). Depression is currently one of the most burdensome diseases worldwide (2, 3) and has become a primary global public health concern with a steadily increasing lifetime risk of depression (4, 5). A complex interplay of social, psychological, and biological factors is involved in the pathogenesis of depression (6). Patient Health Questionnaire-9 (PHQ-9), the most important scale in clinical and population-based research depression screening, has been written extensively in depression guidelines (7, 8). International guidelines recommend screening for depression (9), identifying PHQ-9 as the most reliable screening tool (10). A better understanding of the mechanisms of depression is crucial for its further prevention and treatment.

Moderate levels of thyroid hormones are essential for normal brain development, mood, and cognitive function (11). Thyroid hormones are not only critical in the developing nervous system, but they are also equally important in the adult brain (11). Abnormal changes in thyroid hormone markers can lead to a variety of thyroid dysfunctions such as Hashimoto’s thyroiditis, hypothyroidism, hyperthyroidism, thyroid cancer, and so forth. Currently, the link between depression and thyroid dysfunction is beginning to attract progressive attention. One study showed that hypothyroidism was significantly associated with treatment-resistant depression (12). Another study showed no association between genetically predicted thyroid-stimulating hormone (TSH) or free thyroxine (FT4) and the risk of major depressive disorder (13). In summary, the relationship between depression and thyroid-related hormones remains controversial.

In this study, we conducted a cross-sectional analysis of the relationship between PHQ-9–based depressive symptoms and thyroid hormone in American adults using a large sample of aged 20–80 years from the National Health and Nutrition Examination Survey (NHANES) of 2007–2012. Our findings provided epidemiological evidence to further determine the relationship between depressive symptoms and thyroid disease in adults.

## 2 Materials and methods

### 2.1 Study population and design

NHANES is a population-based national cross-sectional survey conducted by the National Center for Health Statistics of the Centers for Disease Control and Prevention. The NHANES is performed biennially with an extensive multistage cluster design, and the main sections of the database include demographics, dietary, physiological measurements, laboratory tests, and multiple questionnaires. Data from subjects in 2007–2008, 2009–2010, and 2011–2012 were selected for this study, which was the only period that NHANES has collected thyroid function data to date. The NHANES protocol was approved by the National Center for Health Statistics Research Ethics Review Board, and all participants signed informed consent. For more detailed information about NHANES, please visit https://www.cdc.gov/nchs/nhanes/index.htm.

**Figure 1:**
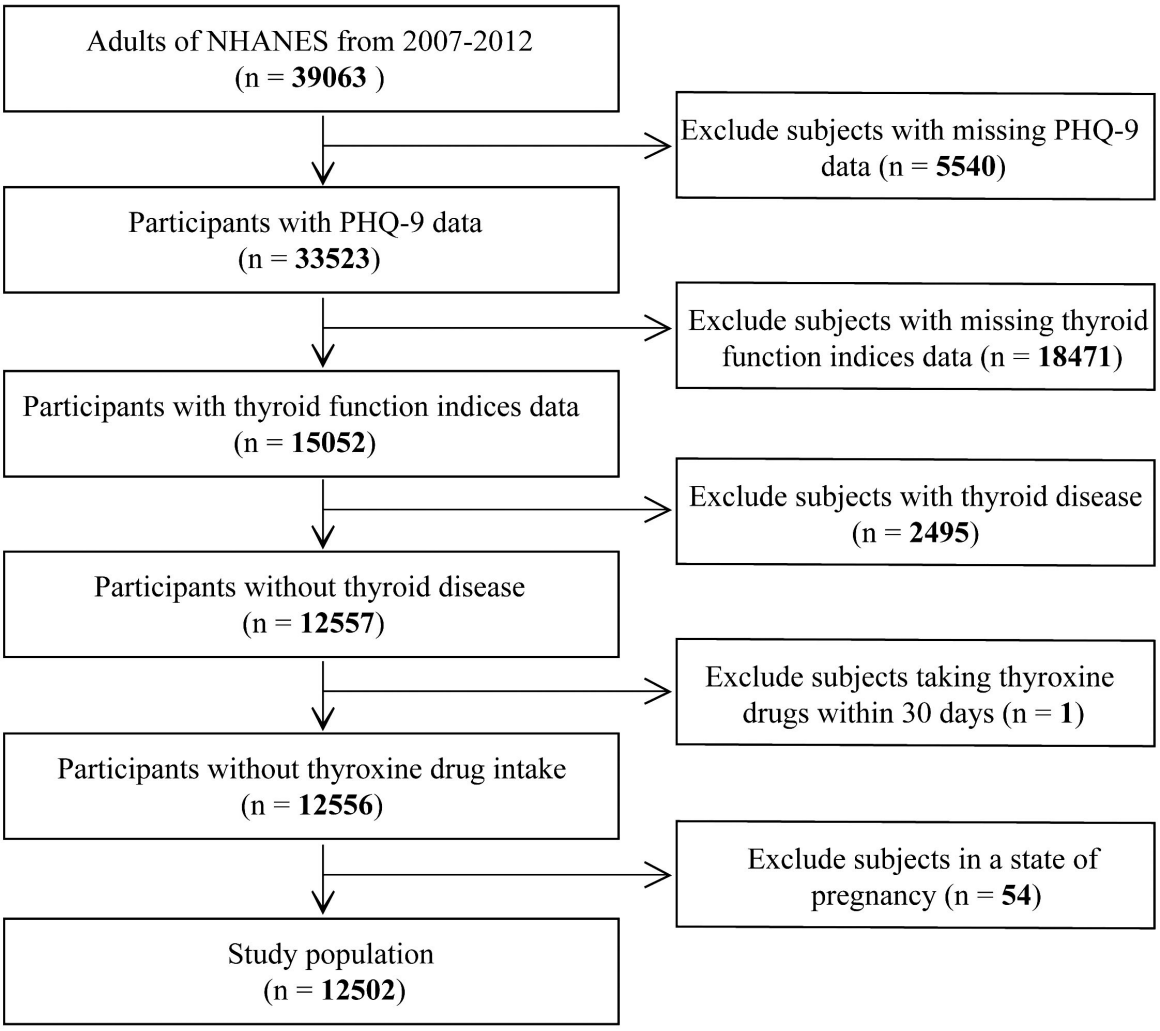
Study flowchart. NHANES, National Health and Nutrition Examination Survey.

A total of 39,063 adults (≥20 years of age) enrolled in the NHANES study from 2007–2012; we excluded the following participants: (1) incompleted PHQ-9 screening scale (*n* = 5540); (2) absence of thyroid function measurement (*n* = 18471); (3) history of thyroid disease diagnosis (*n* =2495); (4) taking a prescription drug that may affect thyroid function in the past 30 days (*n* = 1); (5) pregnant at the time of the survey (*n* = 54). Finally, 12,502 subjects were included (**Figure 1**).

### 2.2 Measurement of PHQ-9

The PHQ-9 is a nine-item depression screening tool that measures depression severity. It is based on the criteria of the Diagnostic and Statistical Manual of Mental Disorders (Fourth Edition) (14). Each item of the PHQ-9 is scored on a scale from 0 (“not at all”) to 3 (“nearly every day”), and the total score ranges from 0 to 27 (15). Based on previous studies, a score of 5–10 represents mild depression, 10–15 represents moderate depression, 15–20 represents moderately severe depression, and a score above 20 represents severe depression (16).

### 2.3 Measurement of thyroid function

Free triiodothyronine (FT3) (pg/mL), total T3 (TT3) (ng/dL), and total T4 (TT4) (µg/dL) were detected by competitive binding immunoenzyme assay, and FT4 (ng/dL) was measured by two-step enzyme immunoassay. TSH (mIU/L) was determined by the third-generation two-site immunoenzyme sandwich method. Antithyroglobulin antibody (TgAb) (IU/mL) and antithyroperoxidase antibody (TPOAb) (IU/mL) were measured by a two-step immunoenzyme sandwich method. Thyroglobulin (Tg) (ng/mL) was measured by simultaneous one-step sandwich method.

### 2.4 Covariates

Covariates were collected by standardized questionnaires or laboratory tests and included age (years), gender (male and female), ethnicity, body mass index (BMI), urinary iodine concentration (µg/L), cotinine level (ng/mL), household income poverty ratio (PIR), and moderate physical activity. Race was categorized as Mexican American, non-Hispanic white, non-Hispanic black, other Hispanic, and other races. BMI was calculated as weight (kg) divided by height squared (m^2^). Subgroups were divided by age: ≥ 60 years old was defined as the elderly population and 20 ≤years old < 60 was defined as young adults (17). PIR was a continuous variable to assess participants’ socioeconomic status. Physical activity was defined as YES (have moderate activity) and NO (no moderate activity) (18). Serum cotinine level was used as a marker of nicotine exposure.

### 2.5 Statistical analysis

Statistical computing and graphics software R (version 4.1.3) and EmpowerStats (version 2.0) were used for statistical research. Baseline tables of the study population were statistically described by subgroups with PHQ-9 < 5 (no depressive symptoms) and PHQ-9 ≥ 5 (with depressive symptoms), and continuous variables were described by mean ± standard deviation (*SD*) and weighted linear regression models. Multiple linear regression was used to analyze beta values and 95% confidence intervals between depressive symptoms and thyroid function indicators. The multivariate test adopts three models: Model 1: no variable adjustment; Model 2: adjust age, gender, and race; and Model 3: all covariates were adjusted. Considering the effects of age and gender on thyroid function, subgroup analysis was performed according to age and gender, and all covariates except themselves were adjusted. In addition, interaction effects analyses were performed according to age and gender. *P* < 0.05 was statistically significant. The weighting method was used to reduce significant volatility in the dataset.

## 3 Results

### 3.1 Baseline characteristics of participants

A total of 12,502 adult participants were included in the study, including 8,672 participants without depressive symptoms and 3,830 participants with depressive symptoms. Adults without depressive symptoms differed significantly from adults with depressive symptoms on all covariates except FT3, TT3, TSH, and TgAb in both groups. In contrast, adults with depressive symptoms were more likely to be female and non-Hispanic white people. Compared to adults without no depressive symptoms, the adults with depressive symptoms had higher TT4, TPOAb, Tg, urinary iodine concentration, serum cotinine concentration, and BMI, whereas age, FT4, annual household income index, and physical activity were low (**Table 1**).

**Table 1:** Weighted characteristics of the study population based on PHQ-9 score.

Characteristics	PHQ-9 score		*P*-value
	PHQ-9 score (< 5)	PHQ-9 score (≥ 5)	
Age (years)	54.313 ± 17.162	50.692 ± 15.333	< 0.00001
Gender (%)			< 0.00001
1	57.503	41.148	
2	42.497	58.852	
Race/ethnicity (%)			< 0.00001
Mexican American	5.928	5.947	
Other Hispanic	4.036	6.538	
Non-Hispanic White	73.430	72.729	
Non-Hispanic Black	11.222	11.324	
Other race	5.384	3.462	
FT4 (ng/dL)	0.806 ± 0.155	0.787 ± 0.151	< 0.00001
FT3 (pg/mL)	3.123 ± 0.464	3.111 ± 0.375	0.18134
TT4 (µg/dL)	7.822 ± 1.565	7.971 ± 1.702	< 0.00001
TT3 (ng/dL)	111.349 ± 23.628	111.511 ± 25.062	0.73092
TSH (mIU/L)	1.975 ± 1.746	1.989 ± 1.247	0.65786
TPOAb (IU/mL)	15.682 ± 77.454	21.437 ± 110.427	0.00091
Tg (ng/mL	16.334 ± 30.359	19.362 ± 84.410	0.00326
TgAb (IU/mL)	6.402 ± 65.545	6.692 ± 82.629	0.83544
Urinary iodine (µg/L)	277.602 ± 1103.842	414.128 ± 2549.270	0.00004
BMI (kg/m2)	29.262 ± 6.533	31.744 ± 8.371	< 0.00001
Cotinine (ng/mL)	50.084 ± 126.815	89.376 ± 145.828	< 0.00001
Poverty income ratio	3.140 ± 1.617	2.331 ± 1.585	< 0.00001
Moderate activities (%)			< 0.00001
1	39.019	34.416	
2	60.981	65.584	

Mean ± SD for continuous variables and % for categorical variables, the *P*-value was calculated by weighted linear regression model or weighted chi-square test, respectively.

PHQ, Patient Health Questionnaire; FT4, free thyroxine; FT3, free triiodothyronine; TT4, total T4; TT3, total T3; TSH; TPOAb, antithyroperoxidase antibody; Tg, thyroglobulin; TgAb, antithyroglobulin antibody; BMI, body mass index.

### 3.2 Association between depressive and thyroid function indicators

Multivariate regression analysis showed that in the unadjusted model, depressive symptoms were negatively correlated with FT3 and FT4, and positively correlated with TT4, TPOAb, and Tg. When model 2 adjusted for major confounding factors, depressive symptoms were still correlated with FT4, FT3, and Tg, and showed a correlation with TT3 and TSH but no longer with TT4 and TPOAb. When model 3 further adjusted all the covariates, depressive symptoms were negatively correlated with FT4, FT3, and TT3, and positively correlated with TPOAb (**Table 2**).

**Table 2:** The association between depression and thyroid function indices.

Characteristics	Model 1^a^	Model 2^b^	Model 3^c^
	β (95% CI) *P-*value	β (95% CI) *P*-value	β (95% CI) *P*-value
FT4	−0.002 (−0.003, −0.001) < 0.00001	−0.001 (−0.002, −0.001) < 0.00001	−0.002 (−0.003, −0.002) < 0.00001
FT3	−0.002 (−0.003, −0.000) 0.03197	−0.003 (−0.005, −0.002) 0.00009	−0.004 (−0.006, −0.002) 0.00005
TT4	0.012 (0.006, 0.018) 0.00012	0.005 (−0.001, 0.011) 0.07940	0.001 (−0.006, 0.007) 0.83342
TT3	−0.017 (−0.106, 0.072) 0.70896	−0.186 (−0.272, −0.101) 0.00002	−0.164 (−0.264, −0.065) 0.00123
TSH	0.005 (−0.001, 0.011) 0.11056	0.008 (0.002, 0.014) 0.00551	−0.001 (−0.007, 0.006) 0.88584
TPOAb	0.431 (0.105, 0.757) 0.00958	0.147 (−0.185, 0.479) 0.38475	0.724 (0.347, 1.101) 0.00017
Tg	0.482 (0.289, 0.675) < 0.00001	0.432 (0.236, 0.628) 0.00002	0.045 (−0.085, 0.174) 0.49955
TgAb	−0.113 (−0.374, 0.149) 0.39902	−0.142 (−0.409, 0.124) 0.29552	−0.187 (−0.491, 0.117) 0.22860

^a^no covariates were adjusted.

^b^age, gender, and race were adjusted.

^c^age, gender, race, BMI, family income-to-poverty ratio, moderate activities, urinary iodine, and serum cotinine were adjusted.

### 3.3 Association between depressive and thyroid function indicators stratified by age

To further validate whether the relationship between depressive symptoms and thyroid function differed in the American adult population, we conducted subgroup analyses. In the stratification analysis of subgroups divided by age, depression was significantly negatively correlated with FT4, FT3, and TT3 in both younger adults (*p* = 0.00122, *p* < 0.00001, *p* = 0.00003) and older adults (*p* = 0.00001, *p* = 0.00004, and *p* < 0.00001). In contrast, depression was significantly negatively correlated with TT4 and Tg in older adults (p = 0.00054, p = 0.00695) and positively correlated in younger adults (p = 0.01352, p < 0.00001). The positive correlation between depression symptoms and TPOAb was only found in younger adults (p = 0.00488). In addition, age was an interaction factor for the correlation between depressive symptoms and FT3, TT4, TT3, TPOAb, and Tg (*p* = 0.0400, *p* < 0.0001, *p* = 0.0301, *p* < 0.0064, *p* < 0.0001) (**Table 3**).

**Table 3:** Subgroup analysis of the association between depression and thyroid function by age and gender.

	FT4	FT3	TT4	TT3	TSH	TPOAb	Tg	TgAb
Subgroup analysis stratified by age								
< 60	−0.001 (−0.002, −0.001) 0.00122	−0.009 (−0.011, −0.006) < 0.00001	0.011 (0.002, 0.021) 0.01352	−0.286 (−0.421, −0.151) 0.00003	0.002 (−0.006, 0.010) 0.59762	0.869 (0.264, 1.474) 0.00488	0.291 (0.166, 0.415) < 0.00001	−0.147 (−0.359, 0.065) 0.17458
≥ 60	−0.002 (−0.004, −0.001) 0.00001	−0.005 (−0.007, −0.002) 0.00004	−0.018 (−0.029, −0.008) 0.00054	−0.524 (−0.669, −0.379) < 0.00001	0.009 (−0.004, 0.022) 0.16297	−0.299 (−0.624, 0.025) 0.07083	−0.391 (−0.674, −0.107) 0.00695	0.056 (−0.679, 0.792) 0.88092
*p* for interaction	0.0986	0.0400	< 0.0001	0.0301	0.3711	0.0064	< 0.0001	0.5571
Subgroup analysis stratified by gender								
Male	−0.001 (−0.002, −0.000) 0.0164	−0.003 (−0.005, −0.000) 0.0204	0.005 (−0.005, 0.014) 0.3409	−0.198 (−0.336, −0.060) 0.0050	−0.007 (−0.017, 0.003) 0.1443	0.110 (−0.440, 0.660) 0.6943	0.019 (−0.169, 0.208) 0.8405	−0.289 (−0.735, 0.156) 0.2031
Female	−0.002 (−0.003, −0.001) < 0.0001	−0.007 (−0.010, –0.005) < 0.0001	−0.007 (−0.016, 0.002) 0.1389	−0.450 (−0.584, −0.315) < 0.0001	0.007 (−0.003, 0.017) 0.1586	0.600 (0.064, 1.137) 0.0282	0.001 (−0.185, 0.183) 0.9916	−0.200(−0.634, 0.234) 0.3660
*p* for interaction	0.2780	0.0146	0.0860	0.0102	0.0422	0.2106	0.8795	0.7787

Analyses were adjusted for age, gender, race, BMI, family income-to-poverty ratio, moderate activities, urinary iodine, and serum cotinine.

### 3.4 Association between depressive and thyroid function indicators stratified by gender

As shown in **Table 3**, the subgroup analyzed by gender revealed that, in the fully adjusted model, depressive symptoms were significantly negatively correlated with FT4, FT3, and TT3 in both adult males (*p* = 0.0164, *p* = 0.0204, and *p* = 0.0050) and adult females (*p* ≤ 0.0001, *p* < 0.0001, *p* < 0.0001), which were more prominent in females. The positive correlation between depressive symptoms and TPOAb was only found in adult females (*p* = 0.0282), while no significant correlation was performed in adult males. In addition, gender was an interaction factor for the correlation between depressive symptoms and FT3, TT3, and TSH (*p* = 0.0146, *p* = 0.0102, and *p* = 0.0422) (**Table 3**).

## 4 Discussion

The purpose of this study was to assess the relationship between depressive symptoms and thyroid function among American adults. In the cross-sectional analysis of 12,502 participants, we demonstrated significant negative correlations between depression with FT4, FT3, and TT3, and significant positive correlations with TPOAb in adults. However, there were no significant correlations with TT4, TSH, TgAb, and Tg. Notably, age was an interaction factor for the correlation between depressive symptoms and FT3, TT3, TT4, TPOAb, and Tg. Gender was an interaction factor for the correlation between depressive symptoms and FT3, TT3, and TSH.

The limbic system, which widely expresses thyroid hormone receptors, is implicated in the pathogenesis of mood disorders (19). The most prominent clinical effects of thyroid hormone either deficiency or excess is manifested in altered central nervous system function, including mood and cognition (6). Minor changes in thyroid hormone levels may significantly impact brain function in depressed patients, even within the normal range (20, 21). Remarkably, in the normal population and patients with clinical thyroid dysfunction, the effects of thyroid hormones on mood may be different (21). Both thyroid dysfunction and depression are common in the clinic (22), but the connection between them is scarce and controversial. It is unclear whether abnormal concentrations of thyroid hormones are a cause or a consequence of depression (23). A study found that among 263 patients with depression, 69 (26.2%) had thyroid dysfunction (24). Conversely, most adults with thyroid dysfunction also experience psychiatric symptoms (25). The study found a positive correlation between depression and T4 and a negative correlation with TSH, and that higher levels of thyroxine within the normal range were associated with an increased risk of depression (21). Another study revealed that, compared with the control group, T3 and TSH levels were significantly reduced in depressed patients, while the difference in T4 levels was not statistically significant (26). Consistent with the above studies, Kumar et al. demonstrated that people with low TSH had a higher incidence of clinically relevant depression (27). Delitala et al. found that there was a U-shaped relationship between FT4 and depressive symptoms, compared to the mean FT4 values, either high or low FT4 was associated with more depressive symptoms (19). The above study suggested that dysfunction of the hypothalamic-pituitary-thyroid (HPT) axis was associated with the pathophysiology of depression. This can guide the clinical selection of early treatment for related organic diseases rather than just psychotherapy.

However, the exact mechanism between the HPT axis and depressive symptoms remains unclear (28, 29). This could be due to several factors. Nocturnal surge of TSH is frequently absent (30–32) and diminished TSH response to thyrotropin-releasing hormone (TRH) (30, 33) in some depressed patients. This suggested that depressed patients may have some degree of central hypothyroidism, which may result in a decrease in thyroid hormone secretion and, therefore, functional central hypothyroidism may occur in some of them. Furthermore, the occurrence and treatment outcome of mental disorders in hypothyroidism patients were related to genetic factors (25). Valuably, it has been found that antidepressant drugs acted differently in various species, resulting in different antidepressant drugs having different effects on thyroid hormone levels (31), which suggested that clinical drug use in patients with depression needs to be more individualized. Therefore, to understand the role of the HPT axis in the pathogenesis and treatment of depression, further prospective studies and epidemiologic studies are needed to better elucidate it.

Thyroid hormones are essential in the developing nervous system, and in the adult brain, the brain-thyroid relationship remains important (25). Research suggested that age may be an influencing factor in thyroid disease and depression, but the results were contradictory. A cohort study showed that older adults with low TSH levels in the normal range had more concurrent depressive symptoms and a significantly increased risk of developing depression in subsequent years (20). A meta-analysis showed that subclinical hypoidism was positively associated with the risk of depression, especially in people over 50 years of age (34). However, another meta-analysis showed that subclinical hypoidism was associated with depression in younger patients (<60 years old) but not in older patients (≥60 years old) (17). We also stratified the analyses by age; the result showed that depression was significantly negatively correlated with FT4, FT3, and TT3 in adults. However, in TT4 and Tg, depressive symptoms were significantly positively correlated with younger patients (<60 years old) and negatively correlated with older patients (≥60 years old). The positive correlation between depression symptoms and TPOAb was only found in younger adults. These differences are worthy of further investigation. Moreover, age was the interaction factor between depressive symptoms and FT3, TT4, TT3, TPOAb, and Tg. Other studies have found gender differences between thyroid function and depression (35). TSH was associated with depression only among adult males, but no association among females (35), which may reflect greater use of thyroid medications and antidepressants in women. In this study, we found that, in the fully adjusted model of stratified analysis based on gender, the negative correlation between depressive symptoms and FT4, FT3, and TT3 was more prominent in adult females. In addition, gender was an interaction factor for the correlation between depressive symptoms and FT3, TT3, and TSH. All the above studies suggested that depression was related to thyroid dysfunction, and age and gender differences were observed.

The autoimmune process of the thyroid itself may cause psychiatric symptoms in vulnerable patients (25). Although there was evidence that depression was not characterized by significant thyroid dysfunction, it may manifest as subtle activation of thyroid autoimmune processes (36). The susceptibility to major depression was increased in autoimmune thyroiditis (AIT) patients with normal thyroid function (37). The reason may be that AIT causes associated hypothyroidism, which is considered a risk factor for depression and may result in ineffective medication (38). Moreover, AIT often coexists with other autoimmune diseases, suggesting that these patients may have inherent abnormalities in immune regulation. It has also been suggested that thyroid antibodies may have a direct effect on brain function (25). In addition, some depressed patients had abnormalities of the HPT axis, elevated T4 and TRH concentrations, and positive antithyroid antibodies (23). TPOAb was positively correlated with the characteristic markers of depression, and the presence of TPOAb may be a susceptibility marker of depression (39). Most depressed patients had a high level of antithyroid peroxidase (anti-TPO) antibody levels, so it may have diagnostic value in AIT and depression (40). There was also an association between anti-TPO levels and symptomatic distress in AIT patients (38). The median concentration of TSH receptor antibody (TRAb) in females with depression was significantly higher than that in the control group, indicating that TSH receptor antibody may be a biomarker of immune dysfunction in depressed patients (41). Notably, the study suggested that thyroid immunity was significantly associated with mood symptoms, and this effect was more pronounced in premenopausal women (42). In this study, we also noted a gender-differential effect in a fully adjusted stratified analysis model based on gender; the positive association between depressive symptoms and TPOAb was only seen in adult females, while there was no significant association between depressive symptoms and TgAb. These studies suggested that adult females may be more susceptible to depression and AIT. The potential link between depression and autoimmune-related diseases such as AIT could be of interest for future research.

Overall, our study was well powered and indicated strong evidence for a link between thyroid and neuropsychiatric function. We used PHQ-9 screening to study the relationship between depressive symptoms and thyroid function indicators and used a total score to evaluate the severity of depressive symptoms: a score of 5–10 represents mild depression, 10–15 represents moderate depression, 15–20 represents moderately severe depression, and a score above 20 represents severe depression (16). The use of a large, reliable sample size and full adjustment for covariates in this study enhanced the credibility of the study and highlighted the association between depression and thyroid disease. Importantly, we found that the mechanism may be autoimmune related in adult females and iodine concentration may be an important interaction factor. Therefore, monitoring thyroid hormone levels in patients with depression is important for the prevention, tracking, and treatment decisions of depression patients.

However, it is not without limitations. First, this was a cross-sectional study, and future prospective studies with larger cohorts are needed to explore causality. In addition, the data were collected from the American NHANES database, and the assessment was limited to the adult population of a single American country sample, limiting ethnic diversity and hindering the generalizability of our findings. In addition, although we have adjusted for covariates as much as possible, other confounding factors such as medication use in depressed individuals may still affect the results and the findings should be interpreted with caution. In summary, our study revealed that depressive symptoms were negatively correlated with FT3, FT4, and TT3 levels and positively correlated with TPOAb levels, and the result was even more evident in adult females. Further studies are needed to validate it.

## Data Availability

The raw data supporting the conclusions of this article will be made available by the authors, without undue reservation.
